# Barriers Experienced by Medical Students in Conducting Research at Undergraduate Level

**DOI:** 10.7759/cureus.4452

**Published:** 2019-04-13

**Authors:** Jai Kumar, Aurangzeb Memon, Ankeet Kumar, Raj Kumari, Besham Kumar, Sundus Fareed

**Affiliations:** 1 Internal Medicine, Liaquat University of Medical and Health Sciences, Jamshoro, PAK; 2 Internal Medicine, Jinnah Sindh Medical University, Karachi, PAK; 3 Internal Medicine, People's University of Medical and Health Sciences for Women, Karachi, PAK; 4 Internal Medicine, Jinnah Postgraduate Medical Center, Karachi, PAK; 5 Internal Medicine, Civil Hospital, Karachi, PAK

**Keywords:** medical students, clinical research, barriers, pakistan

## Abstract

Introduction

Undergraduate medical research is very important not only for scientific learning but also for career progress. However, there are barriers, especially in developing countries, that restrict undergraduate research. This study aims to evaluate the barriers experienced by medical students in conducting research at undergraduate level.

Methods

It was an observational, cross-sectional survey conducted with 687 clinical students of two public medical universities of Pakistan. A self-structured questionnaire consisting of seven items was administered to assess the barriers in conducting research at undergraduate level. Data was processed and analysed through SPSS v 22.0 (IBM Corp., Armonk, NY, USA).

Results

Lack of knowledge as a barrier was identified by 90.68% (n = 623) students. The second most common barrier identified by the students was lack of time (88.79%; n = 610), followed by lack of mentoring as the third most common barrier (85.74%; n = 572). Sub-group analysis showed that lack of knowledge, lack of mentoring, limited data base access, lack of time, and lack of finances were more crucial barriers for female gender (p < 0.05). Only lack of interest was a crucial barrier for male gender (p < 0.05).

Conclusion

A number of barriers need to be addressed in order to enhance students’ participation in clinical research such as lack of interest, funding, and poor availability of research mentors and access to scientific databases to improve participation in clinical research. Substantial amendments in the medical undergraduate curriculum are needed.

## Introduction

Modern medicine and healthcare depend and evolve on the basis of evidences which lead to better understanding of the diseases. It is the main reason why physicians should have keen interest in medical research [1]. Considering the importance of research in understanding diseases in all aspects, undergraduate clinical research is currently more valuable and more desperately needed than before [2]. Involving medical students in clinical research activities at undergraduate level boosts their interest and improves their scientific output as opposed to their peers who are not involved in research. Students who have published articles as undergraduate publish three times more articles later as doctors as compared to those students who were not involved in research at student level [3]. A systematic review by Straus et al. stated that having a medical school publication positively influences academic career choice among medical students [4].

Benjamin in his study stated that students in developing and low-income countries face more difficulties in conducting research than developed countries [5]. Only 1.2% of researches globally come from South Asia, and the situation is ever graver in Pakistan due to lack of physician-scientists [6]. Lack of research training in medical students has been held as the primary reason behind unavailability of undergraduate research. Other barriers can be both professional and personal barriers. Professional barriers may include limited access to information, limited access to equipment, and lack of mentorship. Personal barriers may include inadequate knowledge of research methodology, inadequate statistic skills, and time and financial constraint [7, 8].

There is a lack of data available for barriers among medical students in Pakistan in conducting research. In order to allow our medical students flourish academically and scientifically in comparison to peers from developed country, it is important to identify these barriers.

## Materials and methods

Study design, settings, and participants

It was an observational, cross-sectional survey conducted in two public medical universities of Pakistan in February 2019. Nine hundred and twelve clinical students (3rd-5th year) were invited to participate, 707 students agreed to participate (response rate: 77.5%). Twenty responses were excluded due to incomplete information. Hence, there were 687 students who completed the survey.

Study instrument

A self-administered questionnaire was structured. As a pilot project, 50 students of final year were requested to mention any five barriers that they have faced in conducting research. Total of nine items were identified, of which two were removed because of redundancy and the final questionnaire comprised of seven items. These included lack of mentoring, time constraint, lack of research knowledge, lack of interest, lack of research culture, limited access to database and lack of finances. Participants of the actual survey were requested to randomly select any or all barriers that were relevant to them.

Data analysis

Data was processed and analyzed using SPSS for Windows version 22.0 (IBM Corp., Armonk, NY, USA). Internal consistency of the seven items was calculated using Cronbach alpha. Frequency and percentage for each item was calculated. Sub-analysis was done to assess the correlation of gender on these barriers using chi-square. P value ≤ 0.05 was considered significant.

## Results

The study included 687 participants, of these 521 (75.83%) were females and 166 (24.17%) were males.

**Figure 1:**
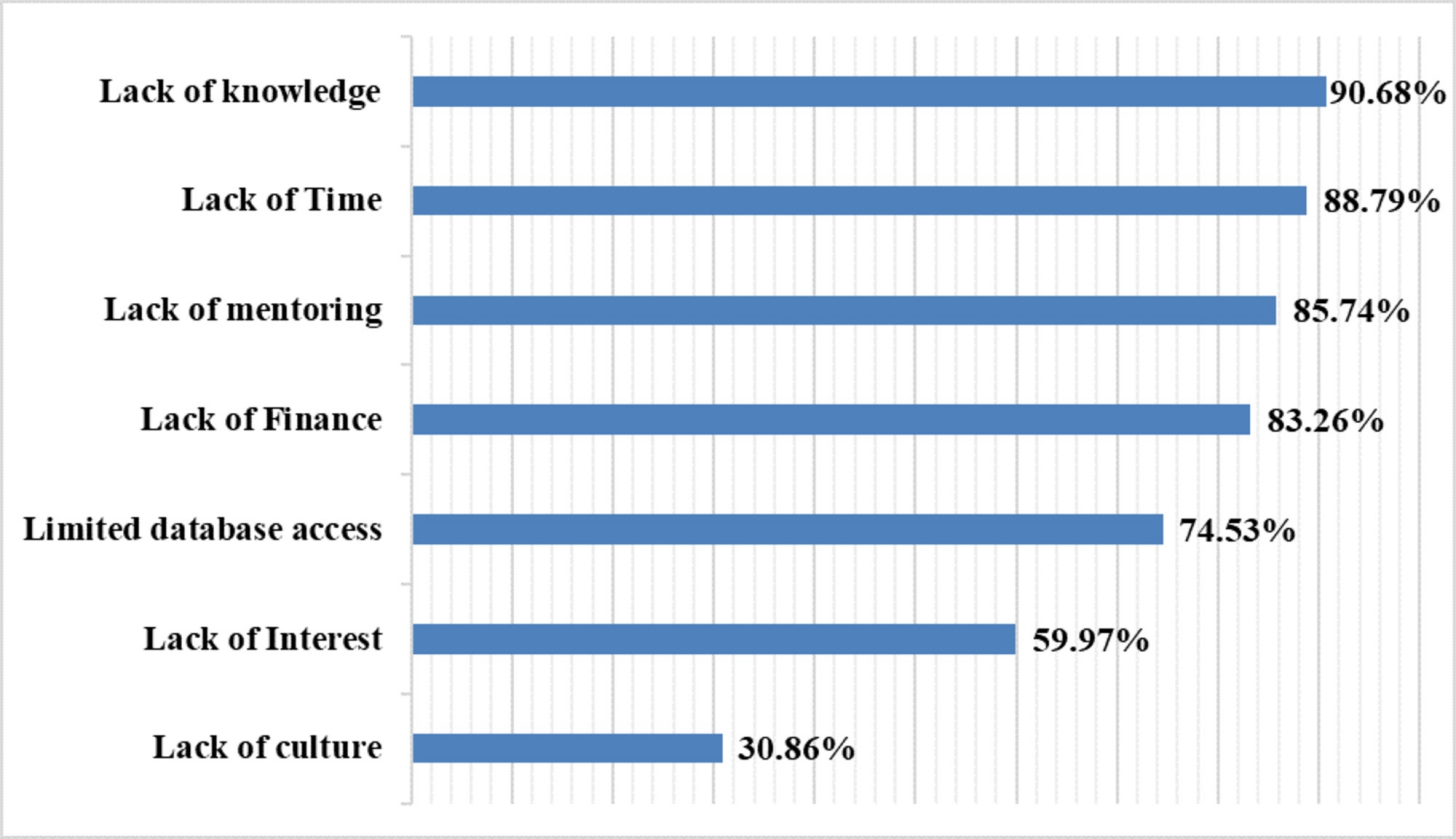
Barriers experienced by medical students in conducting research (n = 687).

The internal consistency of the seven-item scale was 0.78 in this study. Lack of knowledge as a barrier was identified by 90.68% (623 out of 687) students. The second most common barrier identified by the students was lack of time (88.79%; 610 out of 687), followed by lack of mentoring as the third most common barrier (85.74%; 572 out of 687). The common barriers experienced by medical students in conducting research are shown in Figure 1.

Sub-group analysis showed that lack of knowledge, lack of mentoring, limited data base access, lack of time, and lack of finances were more crucial barriers for female gender. Only lack of interest was a crucial barrier for male gender (P value = 0.0001). There was no significant difference in lack of culture barrier as shown in Table 1.

**Table 1:** Impact of gender on the barriers experienced in conducting research (n = 687).

Barriers	Male n (%)	Female n (%)	P value
Lack of knowledge	122 (73.49%)	501 (96.16%)	0.0001
Limited database access	95 (57.23%)	417 (80.04%)	0.0001
Lack of culture	41 (24.70%)	171 (32.82%)	0.05
Lack of mentoring	87 (52.41%)	502 (96.35%)	0.0001
Lack of interest	128 (77.11%)	284 (54.51%)	0.0001
Lack of time	113 (68.07%)	497 (95.39%	0.0001
Lack of finance	129 (77.71%)	443 (85.03%)	0.03

## Discussion

Although, the importance of research is well known and repeatedly stated in the field of medicine, only a small proportion of medical students conduct research [9, 10]. There are many barriers that are responsible for this deficit. In this study, it was seen that major barriers in conducting clinical research as medical students include lack of knowledge, lack of finance, lack of time and lack of mentorship. These results were consistent with the outcomes another study conducted with Egyptian medical school in 2016 [7].

In this study, 90.68% participants stated lack of knowledge regarding research methodology and other aspect of research as their core reason for not conducting clinical research. Bennett et al. also had similar findings regarding lack of knowledge as challenge for research [11]. It is shown that mandatory participation in research activity improves student knowledge [12].

In this study, 88.79% participants stated lack of time as their reason for not conducting clinical research. Lack of time was the major stated reason in a study conducted in Egypt [7]. Similar findings were also stated in Saudi students where 72% of the students consider lack of time as the barrier to conduct research [9]. Siemens et al., in 2010, considered lack of time as a significant barrier in conducting research due to busy academic schedule [10].

In this study, 85.74% of the participants considered lack of mentorship is the main factor stopping them from conducting research. Acquiring new skills such as conducting research is easy when one has a mentor that guides through the learning phase. Mentors not only facilitate in learning skills but also assess progress and advise corrective measures [13]. The results were echoed by similar studies regarding importance of research mentorship for medical students [7, 10].

In this study, lack of finances or funding was considered a barrier by 83.26% participants. Hegde et al. mentioned lack of funding from institutions as the chief barrier for undergraduate research [14].

In this study, limited access to database was also reported as a barrier by more than 59% of the participants. This finding was also consistent in Egyptian medical students [7]. Silva et al. in his study stated that, compared to peers in developed countries, medical students have limited access to database in low income and developing countries [15].

Based on this study, we suggest the need of substantial amendments in the medical undergraduate curriculum. Lack of knowledge was the most common barrier, hence, we recommend interactive sessions and workshops on all aspects of medical research from early undergraduate years. Lack of time was found as a significant barrier against research; therefore, we suggest making research participation mandatory for the students. Research methodology and data analysis should be taught in the classes and it should be included in their final grading. Faculty should mentor the students and encourage them to participate in research activities. In-campus digital access to various databases and online research resources must be provided to the students to boost their interest and streamline the process of conducting medical research for them.

## Conclusions

The major barriers that were experienced by medical students in conducting research in this survey were lack of knowledge, lack of time, lack of mentoring and lack of finance. These hindrances need to be addressed in order to enhance students’ participation in clinical research.
